# Leaving Against Medical Advice From In-patients Departments Rate, Reasons and Predicting Risk Factors for Re-visiting Hospital Retrospective Cohort From a Tertiary Care Hospital

**DOI:** 10.15171/ijhpm.2019.26

**Published:** 2019-05-18

**Authors:** Obada Hasan, Muhammad Adeel Samad, Hamza Khan, Maryam Sarfraz, Shahryar Noordin, Tashfeen Ahmad, Gul Nowshad

**Affiliations:** ^1^ Orthopedic Section, Department of Surgery, The Aga Khan University Hospital, Karachi, Pakistan.; ^2^ General Surgery Department, York Hospital, York, PA, USA.; ^3^ General Surgery Department, Yale University, New Haven, CT, USA.; ^4^ Medical College, The Aga Khan University, Karachi, Pakistan.; ^5^ Department of Biological and Biomedical Sciences, The Aga Khan University Hospital, Karachi, Pakistan.; ^6^ Pioneer Public Health Consultants (PPHCUSA), Houston, TX, USA

**Keywords:** LAMA, DAMA, Morbidity, Developing Country

## Abstract

**Background:** Approximately 1% to 2% of hospitalized patients get discharged or leave from the hospital against medical advice and up to 26% in some centers. They have higher readmission rate and risk of complications than patients who receive complete care. In this study we aimed to determine the rate of leave against medical advice (LAMA) and reasons for the same across different in-patient departments of a tertiary care hospital.

**Methods:** Retrospective cohort study on patients admitted in all departments at our institute over a 1-year period. All patients who were admitted to an in-patient ward at the hospital and who left against medical advice by submitting a duly filled LAMA form were included. Univariate and multivariate logistic regression models with forward selection methods were employed. Revisit to hospital within 30 days; to clinic or emergency department was outcome variable for regression.

**Results:** From June 2015 to May 2016 there were 429 LAMA patients, accounting for 0.7% of total admissions. Females were 223 (52%) compared to males 206 (48%). Finances were quoted as the most common reason for LAMA by 174 (41%) patients followed by domestic problems 78 (18%). Internal medicine was the service with the highest number of LAMA patients ie, 153 (36%) followed by Pediatric medicine with 73 (17%). Of the 429 patients, 147 (34%) patients revisited the hospital within 30 days. Sixty-one percent of these ‘bounced-back’ LAMA patients had worsening or persistence of same problem, or new problem/s had developed. In unadjusted bivariate logistic model, patients who were advised for follow-up during discharge against medical advice were four times more likely to revisit the hospital. Patients who were married had an increased odd of revisiting the hospital.

**Conclusion:** Financial reasons are the most common stated reasons to LAMA. Patients who LAMA are at a high risk of clinical worsening and ‘bouncing back.’ This is the first study from our region on in-patient LAMA rates, to our knowledge. The results can be used for planning measures to reduce LAMA rates and its consequences.

## Introduction

Discharge or leave against medical advice (LAMA) of hospitalized patients is an adverse clinical event often resulting from a fundamental disagreement between the patient or an interested third party and the attending physician and/or the hospital environment.^1^ Approximately 1% to 2% of inpatient stays result in discharges against medical advice,^2^ but may reach up to 25.9% in some centers.^3^ The rate of LAMA also vary with the department which admits the patient. Rates of 6% to 54% for psychiatric admissions and 0.9% for emergency admissions have been reported.^4^ LAMA is a matter of concern because results of many experimental studies have shown that patients discharged against medical advice have more readmission prevalence and also higher risk of complications than patients who receive care completely.^5^ Moreover, lawsuits related to discharges seem more common among those discharged against medical advice. A study conducted by Quinlan and Majoros reported that 0.3% of LAMA cases led to litigation compared to 0.05% caused by regular discharges.^6^

Various factors have been identified as predictors of LAMA. They fall within 2 broad categories: First is patient variables – socio-demographic characteristics, diagnosis, treatment history, behavior; and attitudes toward treatment – and second is provider variables – hospital setting and structure, staffing patterns, admission and discharge policies, and physicians’ clinical style and experience.^7^ Younger age, male gender, poor social support, lack of healthcare coverage, psychiatric illness, drug or alcohol abuse are frequently associated with discharge against medical advice (AMA).^8^ Moreover, those with prior discharge against medical advice have been identified as risk factors for discharge against medical advice.^4^

Previous studies have reported numerous reasons for patients for requesting LAMA. Critically ill conditions of the patient with almost no hopes for survival, dissatisfaction with the treatment provided by the hospital and competing family responsibilities are a few of them.^9^ Some other reasons identified are patients’ expectation of a shorter stay, patient feeling better and preference for another hospital.^10^

The prevalence of LAMA is higher in developing countries as compared to developed countries.^1^ Review of literature revealed only one study identifying reasons of LAMA in Pakistan. It was conducted at a facility in Rawalpindi, Pakistan and included only inpatient psychiatric patients. It identified that frequency of patients who left against medical advice was found to be more in males (63.5%), younger age groups (21-30 years), lesser educated (more than half were under matric) and with the ICD-10 diagnosis of substance abuse (23.9%).^11^ Considering the scarcity of data on LAMA in Pakistan and absence of reports from multiple in-patient departments, we aimed to determine the rate of LAMA and reasons across different in-patient departments of a tertiary care university hospital in Karachi, Pakistan.

## Methods

We conducted a retrospective study on patients at a tertiary care center to assess LAMA from inpatient ward. Institutional ethics committee approval was taken beforehand. Study was conducted by the department of Orthopedics, and all hospital stakeholders were taken into confidence. Inclusion criteria were all inpatients admitted to the hospital and who left against medical advice and filled out the LAMA form June 2015 to May 2016. Patients who LAMA but did not sign the LAMA form were excluded.

Data on gender, age, marital status, day and time of admission and discharge, duration of stay, primary specialty for care, reason for leaving against medical advice, prior history of hospitalizations, mode of payment, medication and follow-up advice at time of LAMA, revisit to the hospital and development of morbidity at follow-up was collected from patient charts and the LAMA form. Medical records were utilized as well as the online health information management system to confirm the readmission and if any revisits to clinic which was not documented in medical record. LAMA form is same for all departments and it is mandatory to be filled and signed by patient or attendant before the patient leaves the ward. It has a section for patients’ comments, as well as pre-specified reasons that patient should choose. It is countersigned by the admitting consultant team which includes medical officer, house officer or on-call resident.

All quantitative data was presented as mean or median. Revisit to hospital within 30 days; to clinic or emergency department was outcome variable for regression. Univariate and multivariate logistic regression models with forward selection methods were employed to assess the combine effect of variables together in the model and to adjust the confounders if present. Analysis was done by one of the senior co-authors with clinical experience. SPSS version 20 and STATA version 13 were used for analysis.

## Results

Total 61 998 patients were admitted during the study period. Patients who opted for to LAMA were 429. Females were 223 (52%) compared to males 206 (48%). Median age of subjects was 45 years ranging from newborns to 98-year-old patients and majority of the participants were married (63%) followed by singles (16%) (Table 1).

**Table 1:** Characteristics of Patient Population

**Characteristics**		**No. (%)**
Age (y)	Median: 38 years	Range: 0-98
Gender	Male	206 (48)
	Female	223 (52)
Marital status	Married	270 (62.9)
	Single	70 (16.3)
	Not applicable	47 (11.0)
	Not written	42 (9.8)
Day of visit	Monday	71 (16.6)
	Tuesday	58 (13.5)
	Wednesday	63 (14.7)
	Thursday	64 (14.9)
	Friday	70 (16.3)
	Saturday	52 (12.1)
	Sunday	51 (11.9)
Day of discharge	Weekday	319 (74.4)
	Weekend	110 (25.6)
Length of stay	Mean (SD)	3.81 days (3.46)
Reason for LAMA	Lack of finances	174 (40.6)
	Reason not specified	65 (15.2)
	Dissatisfied with healthcare	34 (7.9)
	Dissatisfied with physical arrangements	10 (2.3)
	Dissatisfied with ward/inpatient routines	3 (0.7)
	Domestic Problems	78 (18.2)
	Wish to continue treatment elsewhere	42 (9.8)
	Not allowed by payer	2 (0.5)
	Others^a^	21 (4.9)
Method of payment	Self	367 (85.5)
	Employer	21 (4.9)
	Third party	41 (9.6)
Prescribed medicine	Yes	198 (46.2)
	No	231 (53.8)
Advised follow- up	Yes	274 (63.9)
	No	155 (36.1)

Abbreviations: LAMA, leave against medical advice; SD, standard deviation. ^a^Other reasons included feeling better in 8 patients, wants to be managed by my own in 6 patients, want 2nd opinion in 2 patients, security threat, wants to leave hospital, environment, wants to go back home, surgery delayed and has to join job in 1 patient for each.

The percentage of admissions on all the days of the week was similar ranging from 12.0% (Sunday) to 16.6% (Monday) and mean length of hospital stay was 3.8 days (standard deviation: 3.5). Patients who had a history of previous hospitalization were 150 patients (34.96%). Among these 33 (7.7%) had a history of LAMA whereas 117 (27.3%) patients were discharged in stable condition.

Lack of finances was quoted as the most common reason for LAMA by 174 (40.6%) subjects followed by domestic problems 78 (18.2%) which includes the need to take care of dependent children or spouse and other family stress caused by patient’s hospital stay or the need for handling personal affairs at home, reason not specified 65 (15.2%), wish to continue treatment elsewhere 42 (9.8%), dissatisfied with healthcare 34 (7.9%), dissatisfied with physical arrangements 10 (2.3%), dissatisfied with ward routine 3 (0.7%), and not allowed by payer 2 (0.5%). Twenty-one patients (4.9%) reported other reasons, among which ‘feeling better’ most commonly was reported.

Self-payment was the most common method of payment which was reported by 367 patients (85.5%) followed by payment from third party for 41 patients (9.6%) and payment by employer for 21 patients (4.9%).

There was variation in LAMA rate between different departments (Table 2). The department of internal medicine constituted the highest number of LAMA patients: 153 (35.7%) followed by pediatric medicine 73 (17.0%), obstetrics/gynecology 37 (8.6%), and psychiatry 28 (6.5%). General surgery had 16 (3.7%) LAMA patients, which is the highest number recorded among all the subspecialties of surgery.

**Table 2:** Distribution of LAMA Patients in Between All Departments^a^

**Departments**	**Sub-divisions**	**Number of LAMA (% of Total LAMA)**	**Department Admissions (% of Total Admission** ^b^ **)**	**% of LAMA in Each Department**
Medicine	Internal medicine	153 (35.7)	3578 (5.8)	4.28
	Cardiology	11 (2.6)	1733 (2.8)	0.63
	Gastroenterology	15 (3.5)	1672 (2.7)	0.90
	Infectious diseases	9 (2.1)	1546 (2.5)	0.58
	Neurology	8 (1.9)	1225 (2.0)	0.65
	Pulmonology	15 (3.5)	1216 (2.0)	1.23
	Endocrinology	17 (4.0)	1214 (2.0)	1.40
	Nephrology	2 (0.5)	508 (1.0)	0.39
	Acute care unit	10 (2.3)	62 (0.1)	16.1
	Oncology/hematology	15 (3.5)	3159 (5.1)	0.47
Obstetrics/Gynecology		37 (8.6)	8665 (11.4)	0.43
Pediatrics	Neonatology	2 (0.5)	13 489 (22.0)	0.01
	Pediatric medicine	73 (17.0)	10 163 (16.0)	0.72
	Neonatal ICU	1 (0.2)	581 (1.0)	0.17
Psychiatry		28 (6.5)	595 (1.0)	4.71
Surgery	General surgery	16 (3.7)	4087 (6.6)	0.39
	Orthopedic surgery	5 (1.2)	2406 (3.8)	0.21
	Neurosurgery	5 (1.2)	2236 (3.6)	0.22
	Cardiothoracic surgery	1 (0.2)	1232 (2.0)	0.08
	Pediatric surgery	2 (0.5)	1022 (1.6)	0.20
	Otolaryngology	2 (0.4)	776 (1.3)	0.26
	Vascular surgery	1 (0.2)	568 (1.0)	0.18
	Plastic surgery	1 (0.2)	265 (0.4)	0.38
Surgical Departments^c^		70 (16)	21 257 (34)	0.33
Medical Departments^c^		359 (84)	40 741 (66)	0.88

Abbreviations: LAMA, leave against medical advice; ICU, intensive care unit. ^a^ Excluding emergency department and day care units. ^b^ Total inpatient admissions during the study period were 61 998. ^c^ Total number and % of LAMA patients from total hospital admissions for surgical and medical departments is 70 (0.1%) and 359 (0.6%), respectively.

The highest percentage of LAMA patients was recorded in acute care unit (16.1%), followed by psychiatry (4.7%) and internal medicine (4.3%). Neonatology had the lowest percentage of LAMA patients (0.01%). The total percentage of LAMA in subspecialties of surgery was 0.3% and in subspecialties of medicine was 0.9%. At the time of LAMA, almost half of the patients 198 (46.2%) were prescribed medicines and two-thirds of the patients 274 (63.4%) were advised to follow-up in the clinic.

Out of the 429 patients, 147 (34.3%) patients revisited the hospital within 30 days, out of them 114 patients (77.6%) visited the clinics, 24 (16.3%) visited emergency department and 9 (6.1%) were re-hospitalized (Table 3). Eighty-nine (60.5%) patients who revisited developed morbidity (developed new complaints or worsening of the complaints at the time of admission). Out of these 89 patients who developed morbidity, majority had persistence of the same complaint – 49 (55.1%) or worsening of the same complaint – 27 (30.3%) and only 13 (14.6%) patients had new complaints.

**Table 3:** Post LAMA Characteristics

**Revisited hospital in 30 days, No. (%)**	Yes	147 (34.3)
	No	282 (65.7)
**Portal of revisit, No. (%)**	Follow-up in clinic	114 (77.6)
	Visited emergency room	24 (16.3)
	Readmitted	9 (6.1)
**Developed morbidity, No. (%)**	Yes	89 (60.5)
	No	58 (39.5)
**Types of morbidity, No. (%)**	Persistence of same complaint	49 (55.1)
	Worsening of same complaint	27 (30.3)
	New complaint	13 (14.6)

Abbreviation: LAMA, leave against medical advice.

Patients who opt for LAMA were younger (median age 45 years), 63% married and 52% were females. At the time of discharge 82% of these patients were advised for follow-up compared to 54% of those who did not revisited the hospital. Likewise, 58% had been prescribed with medicine among those who revisited the hospital compared to 40% non-visitors respectively (Table 3).

In unadjusted bivariate logistic model, patients who were advised for follow-up during discharge against medical advice were four times more likely to revisit the hospital within 30 days compared to those who were not advised for follow-up. In multivariate robust logistic models after controlling for the demographic variable (age, gender, and marital status in model 2) and addition of financial factors (insurance or mode of payment), this relationship did not change much (odds ratio [OR]: 3.29; *P *< .00, model 4).

Similarly, patients who had third party payer were 2.2 times more likely to revisit hospital within thirty days compared to self-paying patients (OR: 2.2; *P *< .05, model 4). Patients who were married had an increased chance of revisiting hospital within the month (OR: 2.0; *P *< .05 model 2) after controlling for age and gender but in model 4 after taking financial variables into consideration marital status was not statistically significant as a risk factor for revisit (Table 4).

**Table 4:** Logistic Regression Models Predicting Risk Factors for Revisiting Hospital

**Risk Factors for Revisit**	**Model 1**	**Model 2**	**Model 3**	**Model 4**
Advised to follow-up	3.893^a^	3.944^a^	3.476^a^	3.298^a^
Age		0.992	0.992	0.995
Gender		0.965	0.918	0.934
Married		2.023^b^	1.981^b^	1.700^c^
Prescribed medicine			1.536^c^	1.527^c^
Employer paid				0.764
Third party				2.230^b^
Financial reason				0.428^b^
Domestic problems				0.668
Reason unspecified				0.645
Dissatisfied with care				0.261^a^
Constant	0.203^a^	0.188^a^	0.185^a^	0.330^c^
Observations	428	428	428	428

^a^ *P* < .01; ^b^ *P* < .05; ^c^ *P* < .1.

Those with financial constraints had 2.34 (OR: 0.43; *P *< .05) time less likely to revisit the hospital in next 30 days, and patients who expressed their dissatisfaction with the care were about four times less likely to revisit the hospital (OR: 0.26; *P *< .01) compared to those who cited other reasons beside financial or domestic problems.

## Discussion

Discharge against medical advice in hospitalized patients represents a disruption of care, with these patients representing a high-risk population. Relatively little is known about patient factors influencing this process in a developing country like Pakistan so we conducted our study to look at the characteristics of patients who left against medical advice. Our average patient was slightly more likely to be a female (52%) and married (62.9%) with a median age of 45 years but difference is not statistically significant. This is in contrast to previous studies, which found the largest group of patient leaving against medical advice to be the young male substance abuser.^1,2^ One possible reason for this disparity could be that most of these studies assessed patients admitted in the psychiatric units while our study sought to look at patients admitted in all the inpatient departments. Other reason could be due to the fact that, in our country, females are more than males. Moreover, it could be due to the authority in decision-making. As in Pakistan and most of developing countries the husband or eldest son or brother is the decision-maker for the entire family. Discharges from the department of psychiatry in our hospital constituted only 6.5% of our AMA discharges, explaining the underrepresentation of the above-mentioned subset of patient population.

The department of medicine had the largest number of LAMA cases (35.7%). This is possibly due to the fact that our hospital, a large tertiary care hospital in a major metropolitan city has an extensive medical unit comprising of various subspecialties. It is also most likely to admit patients with chronic, serious illnesses requiring prolonged hospitalization and advanced cases which were referred from other hospitals in rural and sub-urban areas. Previous studies have postulated that these patients often refuse to stay in the hospital for a long time^3^ as do patients presenting with non-specific complaints such as chest pain, headache, nausea and vomiting who require extensive workup in an internal medicine unit to rule out more serious conditions.^4^

The most common reason for leaving against medical advice was identified to be financial constraints (40.6%), followed by domestic problems (18.2%). Most of these patients were not covered by health insurance (85.5%). Issues related to high costs of hospitalizations are frequently seen in clinical practice and evidence from research also supports that patients who are uninsured have a higher probability of not completing their treatment.^5,6^ Hospitals that serve low-income populations also have a higher index of AMA discharges,^5,7^ highlighting the role of financial issues in the disruption and non-provision of medical care. This becomes especially important in context of our population where 11.2 million people live below the poverty line.^8^ Further analysis is required to examine the relationship between the variables of health coverage and financial issues patients have. The other reasons for LAMA identified in our study included wishing to continue treatment elsewhere, dissatisfaction with healthcare/ward routines/physical arrangements and feeling better. All of these factors are well recognized in literature.^3,9,10^ Limitation of these causes in the LAMA form is that they are arbitrary and will not help much in guiding hospital policy makers to address these issues to increase patients’ satisfaction. Also needless to say that one LAMA form can’t be applied to all departments and disciplines in hospital as surgical patients may have different reasons and expectations than medical or psychiatric patients.

A cross-sectional study conducted in Iran studying 752 patients who opted for LAMA concluded that among top reasons for LAMA were personal problems and leaving to another facility. Age, gender and place of residence were the predictors.^11^

Patients who LAMA are at a higher risk of developing complications and needing readmissions.^12^ Thirty-four percent of our patients who had left AMA visited the hospital within 30 days, either at clinic or in the emergency department out of which 60.5% of these patients developed morbidity. While this fits into the pattern described by other studies, it also highlights the key issues of a treating physician’s responsibilities towards a patient who wants to leave AMA, and the difficulty in effectively minimizing complications in these patients. While most of the patients were not prescribed medication on discharge (53.8%), the majority (63.9%) were advised follow-up in clinic. The need for medication treatment and follow-up is related to the illness or symptoms that brought the patient to the hospital, but we assumed that patients who were sick enough to be admitted in hospital require medications. Discharge summaries are made by junior doctors including medical officers and house officers. Emphasis were made after this paper on the proper training of these doctors on how to help patients filling the LAMA form as well as on the need to prescribe the required medicines and advice the fellow up visit and explain the signs and symptoms which may necessitate re-admission in hospital or a visit to clinic or emergency department.

LAMA does not reduce the responsibility of the treating doctor towards his patients.

The limitations of our study included its retrospective study design, which necessitated a dependence on patient records. Due to this we are unable to include those patients in our follow-up who might have presented to or been admitted at another hospital following their discharge AMA. Hence the rate of morbidity and readmissions might be higher than that represented in our study. Our study was carried out at a single institution which is a private tertiary care hospital and might not be generalizable to the larger population or public hospitals who treat patients free of cost. However, as it studied all the patients admitted in our hospital, which serves a large patient population, it is able to provide a more comprehensive overview of patients who leave AMA in our patient setting. This will help in better identifying those patients who are likely to leave and in drafting better plans to minimize complications in these patients. Further studies are needed in this under researched topic including case control studies, prospective cohort studies with appropriate follow-up and mixed methodology studies taking into consideration the patients’ factors, thoughts and feelings.

## Ethical issues

Aga Khan University Ethics Review Committee approved the study (Approval No. 4221 5-oct-16).

## Competing interests

Authors declare that they have no competing interests.

## Authors’ contributions

OH and GN contributed to the conception and design of study, data acquisition, drafting the manuscript after analysis. HK, MAS, and MS did the data collection, first data analysis and interpretation of the results. SN and TA critically reviewed the manuscript and results for important intellectual content. All authors read and approved the final manuscript.

## Authors’ affiliations

^1^Orthopedic Section, Department of Surgery, The Aga Khan University Hospital, Karachi, Pakistan. ^2^General Surgery Department, York Hospital, York, PA, USA. ^3^General Surgery Department, Yale University, New Haven, CT, USA. ^4^Medical College, The Aga Khan University, Karachi, Pakistan. ^5^Department of Biological and Biomedical Sciences, The Aga Khan University Hospital, Karachi, Pakistan. ^6^Pioneer Public Health Consultants (PPHCUSA), Houston, TX, USA.

## Key messages

**Implications for policy makers**

- Policy-makers and decision-makers should know the rate and reasons for patients to opt for leave against medical advice (LAMA).
- This knowledge will help in planning better measurements to reduce this phenomenon and its consequences.
- Better hospital resources utilization and patients’ satisfaction.

**Implications for public**

Leave against medical advice (LAMA) is not without a price. There are many catastrophic consequences on morbidity and severity of the pre-existing disease. Patients who LAMA are at a higher risk of clinical worsening and ‘bouncing back.’ Discussing your concerns and factors with your physician and even the hospital administration can lead to a better result.
